# Fine scale structural variants distinguish the genomes of *Drosophila melanogaster *and *D. pseudoobscura*

**DOI:** 10.1186/gb-2006-7-7-r67

**Published:** 2006-07-27

**Authors:** Stuart J Macdonald, Anthony D Long

**Affiliations:** 1 Department of Ecology and Evolutionary Biology, University of California, Irvine, CA, 92697-2525, USA

## Abstract

**Background:**

A primary objective of comparative genomics is to identify genomic elements of functional significance that contribute to phenotypic diversity. Complex changes in genome structure (insertions, duplications, rearrangements, translocations) may be widespread, and have important effects on organismal diversity. Any survey of genomic variation is incomplete without an assessment of structural changes.

**Results:**

We re-examine the genome sequences of the diverged species *Drosophila melanogaster *and *D. pseudoobscura *to identify fine-scale structural features that distinguish the genomes. We detect 95 large insertion/deletion events that occur within the introns of orthologous gene pairs, the majority of which represent insertion of transposable elements. We also identify 143 microinversions below 5 kb in size. These microinversions reside within introns or just upstream or downstream of genes, and invert conserved DNA sequence. The sequence conservation within microinversions suggests they may be enriched for functional genetic elements, and their position with respect to known genes implicates them in the regulation of gene expression. Although we found a distinct pattern of GC content across microinversions, this was indistinguishable from the pattern observed across blocks of conserved non-coding sequence.

**Conclusion:**

*Drosophila *has long been known as a genus harboring a variety of large inversions that disrupt chromosome colinearity. Here we demonstrate that microinversions, many of which are below 1 kb in length, located in/near genes may also be an important source of genetic variation in *Drosophila*. Further examination of other *Drosophila *genome sequences will likely identify an array of novel microinversion events.

## Abstract 2

Comparative genomics reveals fine-scale structural variants, including microinversions, distinguishing two diverged Drosophila species

## Background

A major aim of comparative and population genomics is to elucidate the inter- and intraspecific genetic variation that contributes to phenotypic change. Understandably, the community has focused on the most common source of genetic variation, substitutions at the nucleotide level [1,2]. However, any catalog of genetic variation is incomplete without an examination of other, potentially more complex, forms of sequence-level variation, for example, large insertions and deletions of DNA, rearrangements, and translocations. Such events have been shown to be important in human disease susceptibility [3,4]. Using the tremendous genomic resources available for humans and chimpanzees, recent work has characterized the pattern of large deletions segregating within the human genome [5-8], polymorphic inversions in humans [5,9], as well as structural genome differences between humans and chimps [9,10].

Traditionally, species of the *Drosophila *genus have been an important system for examining variation in chromosome structure. This is largely due to the ability to directly observe such variation from the banding patterns of salivary gland polytene chromosomes [11]. As a consequence of this technique it has been shown that large paracentric inversions - those that do not include the centromere - frequently segregate in *Drosophila *species [12,13]. Since inversions can become fixed during evolution, they can also drive architectural differences between the genomes of diverged species. The species *D. melanogaster *and *D. pseudoobscura *diverged 25 to 55 million years ago [14], and comparative analysis of the sequenced genomes of the two species has shown radical shuffling of regions within orthologous chromosome arms, likely via a series of overlapping paracentric inversions [15]. Similar observations have also been made in comparisons of other *Drosophila *species [16-18].

Most of the work on *Drosophila *inversions has examined those large events, much greater than a megabase in length, that disrupt chromosome colinearity and gene order. Nevertheless, very small paracentric inversions (below a few kilobases in length) that do not affect gene order may also be common in *Drosophila*. Indeed, Negre *et al*. [19] recently demonstrated the existence of such microinversions in the *Drosophila *genes *labial *and *proboscipedia*. Here, we re-examine the *D. melanogaster *and *D. pseudoobscura *genomes to identify fine-scale structural differences between the species. Using a gene-by-gene sliding window BLAST strategy we identify 95 large insertion/deletion events, the majority of which represent insertions of transposable elements into one of the two genomes. We also identify 143 microinversions, 77.6% of which are below 1 kb in size. Sequence conservation within the microinversion is high (74.9%), suggesting they may harbor functional elements. Since we find microinversions in introns and immediately upstream and downstream of transcribed regions, it is plausible that microinversions act as regulators of alternative splicing and gene expression. Our analyses further confirm the role of inversions as an important source of genome variation in *Drosophila *evolution, showing that inversions in *Drosophila *can act to rearrange sequences at a sub-genic level.

## Results and discussion

Using the genome sequences of the two fruitfly species *D. melanogaster *[20,21] and *D. pseudoobscura *[15], we identified 11,011 orthologous gene pairs. This is not inconsistent with the 10,516 orthologs identified by Richards *et al*. [15]. For each orthologous pair, using a sliding-window framework we BLASTed overlapping, short 31 base-pair (bp) fragments of the *D. melanogaster *gene sequence against the *D. pseudoobscura *ortholog. Recording the details of each BLAST hit allowed fine-scale structural changes (inversions, insertion/deletion events) occurring since the separation of the *D. melanogaster *and *D. pseudoobscura *lineages to be identified.

The bulk of transcribed DNA sequence in *Drosophila *does not code for protein, and may diverge rapidly between species. As *D. melanogaster *and *D. pseudoobscura *are diverged by 25 to 55 million years [14], many transcribed regions may show generally low sequence conservation. In such cases, the power of any approach to detect fine-scale structural variation will be limited. Although a pairwise whole-genome alignment of *D. melanogaster *and *D. pseudoobscura *is available, just 48% of bases can be reliably aligned [15]. Hence, to be confident that tested pairs of sequences are identical by descent, we examined only transcribed regions showing clear evidence for orthology. For analysis we retained 5,738/11,011 (52.1%) conserved orthologous gene pairs (see Materials and methods and Additional data file 1). These orthologs span 42.2 Mb of sequence in *D. melanogaster*, which represents 35.6% of the 118.4 Mb release 4.2.1 *D. melanogaster *genome sequence.

### Intragenic insertion/deletion events

We detected 95 large, intronic insertion/deletion events (indels) distributed across 86 of the 5,738 (1.5%) orthologous gene pairs: 80 genes have a single indel, three genes have two indels, and three genes have three indels (Additional data file 2). Since the 5,738 genes span 42.2 Mb of sequence in *D. melanogaster*, this suggests the rate of large insertion/deletion events is around 2.3 per Mb. The observed number of indel-harboring genes on each of the five major *Drosophila *chromosome arms is not significantly different from expectation (Table 1). The size of the inserted sequence ranges from 1,372 bp to 46,889 bp (mean 7,869 bp; standard deviation (SD) 7,347 bp), and 79/95 (83.2%) of the indels have the insertion in the *D. melanogaster *genome.

Large insertion/deletion events distinguishing orthologous genomic regions can indicate the presence/absence of transposable elements (TEs) [22]. To examine whether the indels we detect represent insertions, we used TE annotations for the *D. melanogaster *genome sequence [23,24], and also compared insert sequences against *Drosophila *TE sequences using BLAST (see Materials and methods for details). Of the 79 indel events showing the insertion in the *D. melanogaster *genome, 70 (88.6%) map to an annotated TE, and 69 of these also BLAST against known *Drosophila *TE sequences. For those indels where the insertion is in the *D. pseudoobscura *genome, 6/16 (37.5%) insertion sequences BLAST to TEs. Since *D. pseudoobscura *TEs are less well curated than those of *D. melanogaster*, it is possible that some/most of the remaining ten indels with insertions in *D. pseudoobscura *are also TEs. Thus, the majority of the indels we identify likely represent TE insertions.

In our analysis we detect TEs indirectly, and in an unbiased fashion, via the identification of large indels. Hence, our observation that the majority of indels have the insertion in the *D. melanogaster *genome suggests that *D. melanogaster *introns harbor more TEs than *D. pseudoobscura *introns. This corroborates the finding of Caspi and Pachter [22] that most of the identifiable TEs in a four *Drosophila *species genome alignment are present solely in the *D. melanogaster *lineage, and represent recent insertions in this species. Given these results, we might suspect that the size of orthologous introns would be greater in *D. melanogaster *than in *D. pseudoobscura*. Indeed, while the lengths of orthologous introns are highly correlated between these species [25], there is a very slight skew towards larger introns in *D. melanogaster *(see supplemental Figure S1 of Richards *et al*. [15]). However, Yandell *et al*. [25] note that while some orthologous introns with highly divergent lengths in the two species may be due to TE insertions (validated by results presented here), most of the differences in the size are subtle and not easily explained by transposons.

### Microinversions

**Figure 1:**
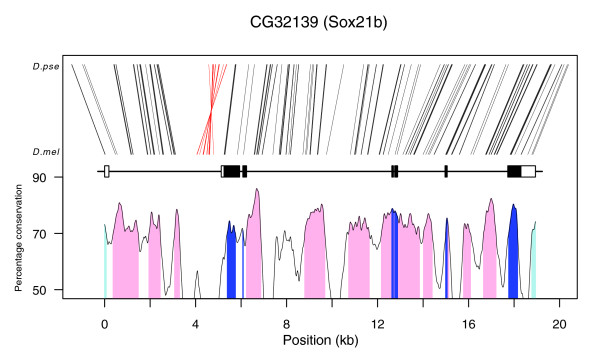
Sequence similarity between *Drosophila melanogaster *(*D. mel*) and *D. pseudoobscura *(*D. pse*) for the *Sox21b *(CG32139) gene. Top panel: sliding-window BLAST profile. We stepped through *D. melanogaster Sox21b *gene in 15 bp increments, and at each position BLASTed a 31 bp segment against the *D. pseudoobscura *ortholog. Each line represents a BLAST hit with a score above 45, the endpoints show the position of the hit in each genome, and the color of the line represents the orientation of the hit (black = same sequence orientation in each genome, red = different orientations in each genome). Central panel: structure of the *Sox21b *gene in *D. melanogaster*. Filled boxes represent exons, and open boxes represent untranslated regions (UTRs). Bottom panel: VISTA plot. The appropriate region of the *D. melanogaster*-*D. pseudoobscura *genome alignment was downloaded from the VISTA Browser [44]. We stepped through the alignment in 5 bp increments, and for each 501 bp window calculated the percentage of identical nucleotides between the sequences. The plot is shown relative to the *D. melanogaster *sequence, and represents a smoothed curve through the data using the ksmooth function in the statistical programming language R [49]. Areas under the curve are painted if they show >70% nucleotide conservation (dark blue = within an exon, light blue = within a UTR, pink = intronic and >100 bp in size).

We detected 121 small inversions within 93/5,738 (1.6%) orthologous gene pairs: 75 genes harbor a single inversion, 10 genes have two inversions, six genes have three inversions, and two genes have four inversions (Additional data file 3). On average, there are 2.9 microinversion events per Mb of transcribed sequence, suggesting that the rate of microinversion may be similar to the rate of large insertion/deletion - primarily TE insertion - events (2.3 events/Mb, see above). One of the intragenic inversions (CG31481_inv1) corresponds to the single *D. melanogaster*-*D. pseudoobscura *microinversion detected by Negre *et al*. [19] in the *proboscipedia *gene. The top panel of Figure 1 shows an example of a typical sliding-window BLAST profile, highlighting an inversion event. One possibility is that the events we identify as microinversions are in fact the result of genome assembly artifacts. To rule this out, three of the inversions (CG3578_inv1, CG3936_inv4, and CG32139_inv1) were confirmed by PCR/resequencing of the inversion breakpoints in both *D. melanogaster *and *D. pseudoobscura*. Also, for each of the 54 intragenic microinversion events less than 500 bp in size in both species we BLASTed the putatively inverted sequence, including 100 bp flanking each breakpoint, against databases of shotgun sequencing reads. When the orientation of the inversion observed in the assembled genome (relative to flanking sequences) is preserved in one or more reads, we can be confident that the microinversion events we detect are not due to errors in genome assembly. Over the 54 inversions, 51 (94.4%) correctly BLAST to at least one read for both species, and on average, inversions correctly BLAST to 10.0 (6.6) sequence reads in *D. melanogaster *(*D. pseudoobscura*). There were no BLAST hits to reads with sequences inconsistent with the inversion orientation in the genome assembly. We conclude that the microinversions we detect are likely real, and not caused by genome assembly artifacts.

Given our success identifying intronic microinversion events, we sought to examine those regions flanking the 5,738 conserved orthologs for microinversions that potentially disrupt upstream or downstream regulatory domains. We extended the sequence of each ortholog by 2 kb upstream and downstream in both *D. melanogaster *and *D. pseudoobscura*, and repeated our sliding-window BLAST procedure. In comparison with our scan of intragenic regions, an analysis of short regions flanking genes has lower power to detect microinversion events for three reasons. First, intergenic sequence is generally less conserved than transcribed intronic sequence, although this difference may be slight [26]. Second, we only scan 2 kb regions, and thus can detect only microinversions below this size. Finally, outside of transcribed regions synteny between the two genomes can break down. Richards *et al*. [15] report that the average number of genes within a *D. melanogaster*-*D pseudoobscura *syntenic block is 10.7, or around 83 kb of sequence. Thus, the intergenic regions we compare may not always be orthologous.

We discovered 22 microinversions in the 19.7 Mb of unique intergenic sequence tested, or 1.1 events/Mb (Additional data file 4). This is proportionally far fewer inversions than we found in intragenic regions (121 microinversions were detected in 42.2 Mb of transcribed sequence, or 2.9 events/Mb), for the reasons stated above. Three of the 22 microinversions were upstream or downstream of genes also harboring an intragenic microinversion event. In total, over both of our sliding-window BLAST tests, we identify 143 unique microinversions distinguishing the genomes of *D. melanogaster *and *D. pseudoobscura*. These 143 events are in/near 112 different genes.

In *D. melanogaster *the frequency of nested genes, genes residing within introns of other genes, is around 7%, and the frequency of overlapping genes is around 15% [27]. None of the microinversions overlap a host gene exon, but 7/143 microinversions overlap an annotated exon from a nested/overlapping gene in *D. melanogaster *(Additional data files 3 and 4). These seven microinversions were not identified by direct scanning of the nested/overlapping genes, presumably due to low sequence conservation of these genes between *D. melanogaster *and *D. pseudoobscura*. It is unclear what, if any, effect these seven microinversions may have on the ability of the orthologous *D. pseudoobscura *nested/overlapping genes to function correctly. To verify that the inverted sequences are single-copy in each of the tested genomes, we BLASTed the sequence of all 143 microinversions against the appropriate genome assembly. The sequences of 142/143 are single copy, while the remaining intronic inversion, CG1794_inv1, BLASTs six times to the genomes of both *D. melanogaster *and *D. pseudoobscura*. The inverted region in this case encompasses the cytosolic tRNA gene *tRNA:met3:46A *(CR30003) that resides in an intron of the *Matrix metalloproteinase 2 *(*Mmp2*) gene. We detect multiple BLAST hits for this sequence because tRNA genes are present in multiple copies throughout the fly genome.

The size of the 143 microinversions ranges from 46 bp to 4,006 bp (mean 628 bp; SD 635 bp) in *D. melanogaster*, and from 40 bp to 4,408 bp (mean 706 bp; SD 731 bp) in *D. pseudoobscura*. The difference in length between the species is due to insertion/deletion of nucleotides. There does not appear to be any strong directional change in microinversion length between the species, as the *D. pseudoobscura *arrangement is longer in just 86/143 (60.1%) of cases. Overall, the majority of microinversions are below 1 kb in both species (111/143, 77.6%). Using Clustalx version 1.83.1 [28,29] we aligned each *D. melanogaster *inversion event sequence with the corresponding, reverse complemented *D. pseudoobscura *sequence. Over the 143 events, ignoring alignment gaps, the average percent nucleotide identity is 74.9% (SD 12.8%). We expect a high level of conservation for the identified microinversions, as our ability to detect them was contingent on sequence conservation. Within the *D. melanogaster *and *D. pseudoobscura *genome alignment, only 46% of the *D. melanogaster *bases are identical [15], and this may generally obscure the signature of historical inversion events. Thus, the 143 detectable, conserved microinversions likely represent only a fraction of the events that have occurred since the divergence of *D. melanogaster *and *D. pseudoobscura*. Comparing the genomes of more closely related species of *Drosophila *may reveal much greater numbers of microinversions.

**Figure 2:**
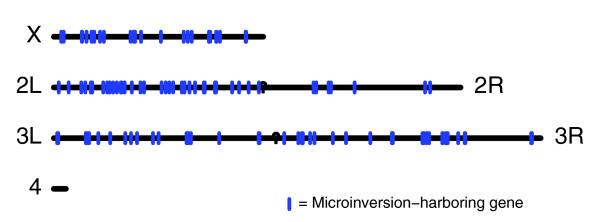
Positions of the 112 microinversion-harboring genes in the *D. melanogaster *genome. Using data from release 4.2.1 of the *D. melanogaster *genome assembly, the physical position of each of the 112 microinversion-harboring genes is mapped onto the *D. melanogaster *chromosomes. The midpoint of each gene is used to map to chromosome. The centromeres for chromosomes 2 and 3 are represented by filled black circles, and the positions of microinversion-harboring genes are indicated by vertical blue lines.

In total, 112 genes harbor a microinversion within the transcribed region or just upstream or downstream. From Table 1 it is clear there is a significant excess of genes with microinversions on *D. melanogaster *chromosome *2L *(Binomial test, *P *= 0.003), and a significant dearth on chromosome *2R *(Binomial test, *P *< 0.001). What is not clear is why this might be the case, as within the major chromosome arms genes containing microinversions appear to be evenly distributed (Figure 2). If we consider the position of the intragenic microinversions within the host genes, they appear to preferentially reside within larger introns. Of the 121 intragenic microinversions, 82 (67.2%) are within the largest host gene intron, and 104 (85.2%) are within one of the largest two introns. Similar values are found when considering only those genes with greater than four introns (data not shown). However, within the host intron, the inversions show no positional preference: over the 121 intronic inversions, the distribution of the distance between the inversion breakpoints and the flanking exons (weighted by the size of the host intron) is approximately uniform (Additional data file 5). These observations are particularly interesting in light of the recent observation that longer introns diverge more slowly than shorter introns in *Drosophila *[30]. If longer introns are under selective constraint, they may be expected to contain many functional motifs, which could be disrupted and/or shuffled around by an intronic microinversion event.

### Impact of microinversions on gene regulation

Comparative genomics seeks to identify functional elements by examining the pattern of sequence conservation across species. The rationale behind this approach is that over evolutionary time sequences will diverge, unless they are under some form of functional or selective constraint. Thus, the maintenance of sequence conservation despite inversion makes the microinversion events we describe particularly interesting, as they may be enriched for functional motifs. Since the microinversions are present both within introns and upstream of genes, this brings up the possibility that inversions might impact the regulation of splicing and gene expression. For example, shuffling transcription factor binding sites within regulatory domains could alter the ability of sets of factors to bind in a coordinated fashion, and thereby up- or down-regulate expression, or alter the timing or tissue-specificity of transcription.

We examined the position of the 143 microinversion events we identify relative to annotated regulatory regions in the *D. melanogaster *genome. We used two complementary resources: the DNase I footprint database is a systematically curated set of 1,362 *Drosophila *transcription factor binding sites [31,32], and the REDfly database is a comprehensive collection of 628 known *cis*-regulatory modules (CRMs; sequences sufficient to regulate gene expression) in *D. melanogaster *[33,34]. None of the DNase I footprints overlap the sequence of any *D. melanogaster *microinversion. However, three microinversions are present within a CRM. Microinversion CG31481_inv1, initially detected by Negre *et al*. [19], resides in intron 2 of the gene *proboscipedia *(*pb*), and is present within a 10.4 kb sequence showing enhancer activity [35]. Microinversion CG1030_inv1, situated just 3' of the gene *Sex combs reduced *(*Scr*), is present within a 6.7 kb region exhibiting enhancer activity [36]. Finally, the inversion CG12287_inv1 resides in intron 3 of the gene *POU domain protein 2 *(*pdm2*), and overlaps a 1.3 kb enhancer region detected and validated by Berman *et al*. [37].

Of course, we do not know whether the microinversions we identify actually have an effect on transcriptional regulation in the two species. It is possible that in the three cases we describe the microinversions have no impact on the spacing/ordering of transcription factor binding sites. This may be particularly true for the two large enhancer regions, which at 10.4 kb and 6.7 kb likely do not represent the minimal enhancer. Work on the *Sox21b *gene, which shows a microinversion in intron 1 (Figure 1), has demonstrated that the pattern of *Sox21b *embryonic expression is conserved between *D. melanogaster *and *D. pseudoobscura *[38]. Thus, for this gene at a particular stage in development, the transcribed microinversion appears to be neutral with respect to expression pattern. As the community begins to understand more about binding site biology and the gene regulatory 'code', we may also be able to determine if the inversions we identify generally have a significant impact on gene regulation.

### Genomic signature of microinversions

In analyzing the breakpoints between the syntenic blocks of *D. melanogaster *and *D. pseudoobscura*, Richards *et al*. [15] provided evidence for a *D. pseudoobscura*-specific breakpoint motif, which could in principle effect large inversions via ectopic exchange. The motif is virtually absent from intron sequences, and is thus unlikely to be the cause of the microinversion events we describe here. In bacteria, short (12 to 23 bp) inverted repeat elements have been shown to permit inversion of the intervening DNA segment [39]. However, the precise mechanism by which very small inversion events occur in eukaryotes in unknown.

**Figure 3:**
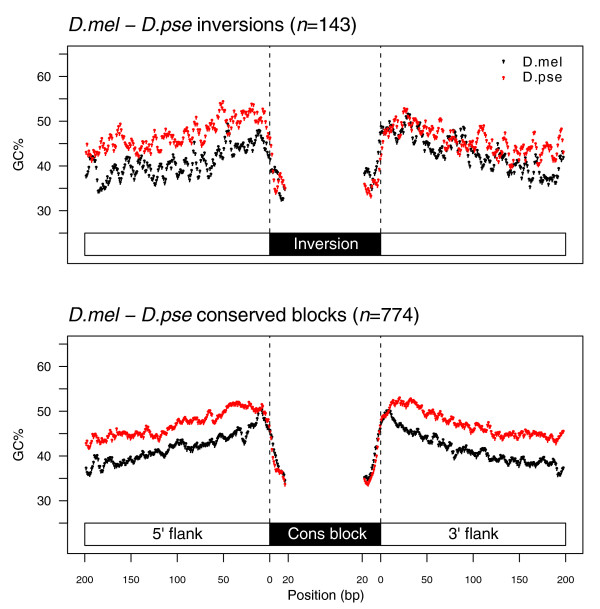
GC content across microinversion breakpoints and conserved sequence blocks. Top panel: 143 *Drosophila melanogaster*-*D. pseudoobscura *microinversions. For each microinversion breakpoint we extracted 200 bp flanking the breakpoint and 20 bp internal to the inversion as a contiguous section (we examined just 20 bp internal to each inversion breakpoint as the minimum inversion size was 40 bp). For each species, across all sequences for a given inversion breakpoint, we calculated GC content for all overlapping 5 bp windows. Each point in the plot represents the mean GC content for a single window. Vertical dashed lines indicate the inversion breakpoints. Note that the distance between these lines is variable across inversion events. Bottom panel: 774 *Drosophila melanogaster*-*D. pseudoobscura *conserved non-coding blocks. Using sliding-window BLAST data we identified all blocks of conserved non-coding sequence from the 93 genes harboring intronic microinversions (see Materials and methods for details). Sequence data were extracted from in/around the conserved blocks and analyzed as described for the microinversion data.

As an initial investigation into this problem, we examined whether the DNA sequence about the microinversion events showed any detectable signature. Richards *et al*. [15] noted that breakpoint junctions between syntenic blocks of *D. melanogaster *and *D. pseudoobscura *were AT rich. The top panel of Figure 3 shows data from a sliding-window analysis of average GC content across the flanking regions and breakpoints for the 143 *D. melanogaster*-*D. pseudoobscura *microinversions. It is apparent that in both species, GC content in the flanking region increases slowly towards the inversion breakpoints, and drops dramatically in the first/last 20 bp of the inversion. The average GC content for introns (where we identify most microinversions) is 40.0% in *D. melanogaster *and 44.0% in *D. pseudoobscura*, and 200 bp from the microinversion, GC content returns to this genome-wide average. Also, the GC content of the inversions themselves is similar to the intronic average (the average GC content for *D. melanogaster *inverted sequence is 42.5%, and for *D. pseudoobscura *is 44.6%).

One possibility is that the GC content pattern we observe across microinversion breakpoints is due not to inversions *per se*, but instead to a change in GC content between conserved and non-conserved sequence: the microinversions we detect essentially represent conserved sequence, present in opposite orientation in the two genomes. We extracted sequence from all 774 conserved non-coding sequence blocks in the 93 genes harboring intronic microinversions (see Materials and methods for details), and subjected these to the same sliding window GC content analysis we performed for the microinversions. As shown in Figure 3, the pattern of GC content across microinversion breakpoints (top panel), and the pattern across junctions between conserved and non-conserved sequence (bottom panel), is identical. The GC content patterns across conserved *Drosophila *sequence are very similar to those recorded by Walter *et al*. [40] for 1,373 blocks of non-coding sequence conserved between human and *Takifugu rubripes *(Fugu). The fact that the pattern is maintained across vertebrate and invertebrate systems is deserving of further work.

In an attempt to distinguish microinversions from conserved blocks based on nucleotide sequence data, we investigated the frequency of all 5-mer sequence motifs across the boundaries of the events, and examined the nucleotide compositional bias at the edges of the events [41]. Neither test clearly distinguished microinversions from conserved blocks (data not shown), suggesting that if there is a general mechanism underlying *Drosophila *microinversion, it is not easily discernible from primary sequence data alone.

### Phylogenetic distribution of microinversion events

**Figure 4:**
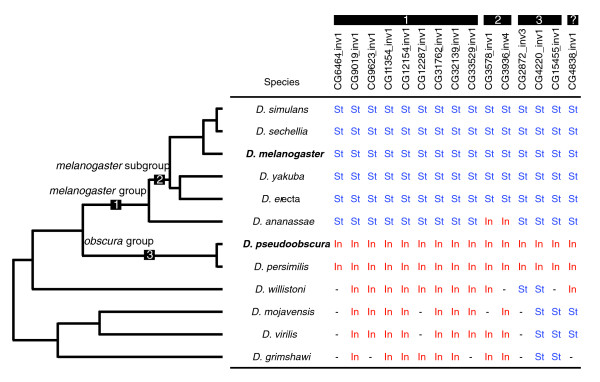
Phylogenetic distribution of fifteen microinversion events. For 15 microinversions distinguishing the genomes of *D. melanogaster *and *D. pseudoobscura*, we examined orthologous regions from 10 other *Drosophila *species to determine whether they harbor the standard (St; *D. melanogaster*-like) or inverted (In; *D. pseudoobscura*-like) arrangement. Some species could not be reliably shown to have either arrangement (shown with a dash). A consensus phylogeny of the 12 species is provided. The microinversion events are grouped according to phylogenetic position, and mapped onto the consensus phylogeny.

It is of interest to ask when the microinversions we identify occurred in the *Drosophila *lineage, and which arrangement (standard or inverted) is the ancestral state. Using data from the 12 recently sequenced *Drosophila *genomes [42] we extracted the orthologous regions surrounding 15 of the intragenic microinversions. For each region we then performed the same sliding-window BLAST procedure we describe above, in each case testing the *D. melanogaster *and the *D. pseudoobscura *orthologs individually against each of the other 11 species' orthologs. Figure 4 details the results of these analyses.

For nine of the events (CG6464_inv1, CG9019_inv1, CG9623_inv1, CG11354_inv1, CG12154_inv1, CG12287_inv1, CG31762_inv1, CG32139_inv1, and CG33529_inv1) the data are consistent with the inversion occurring prior to the divergence of the *melanogaster *group of species. For two events (CG3578_inv1 and CG3936_inv4) the inversion likely occurred prior to the divergence of the *melanogaster *subgroup of five species. Three microinversion events (CG2872_inv3, CG4220_inv1 and CG15455_inv1) occurred along the *obscura *group lineage. Finally, one event (CG4838_inv1) shows the inverted arrangement in the three species *D. willistoni*, *D. persimilis*, and *D. pseudoobscura*, and the standard arrangement in the remaining nine species. Three explanations are compatible with the phylogenetic distribution of CG4838_inv1. First, the same inversion may have occurred independently in the lineage leading to *D. willistoni *and in the lineage leading to the *obscura *group species. Second, the inversion may have occurred prior to the divergence of *D. willistoni *and the *obscura *group species, but re-inverted again in the lineage leading to the *melanogaster *group of species. Alternatively, the state of the CG4838_inv1 microinversion in *D. willistoni *may not be correct, and the inverted form may actually be present only in the pair of *obscura *group species. The latter possibility is conceivable as the current draft assembly of the *D. willistoni *genome has not been subject to the same scrutiny as the genomes of *D. melanogaster *and *D. pseudoobscura*.

Due to ascertainment bias (the microinversion must distinguish *D. melanogaster *and *D. pseudoobscura*) we identify only a particular subset of *Drosophila *microinversions. It will be extremely interesting to extend our analyses to all pairs of *Drosophila *species, and place identified microinversions on the *Drosophila *phylogeny. We predict that many more microinversions will be identified between other *Drosophila *species pairs, and show different phylogenetic patterns.

Finally, we note that the presence of both the standard and inverted arrangements of the 15 tested microinversions in multiple species provides independent support that microinversions are real features of *Drosophila *genome architecture.

### Using BLAST to examine genome architecture

A widely used method to examine sequence differences between/among diverged species is to use VISTA plots of aligned sequence data [43]. This highly informative method allows the local nucleotide conservation between species to be assessed, and VISTA plots can be generated for arbitrary regions of aligned genomes using a web-based utility [44]. However, while the combination of genome alignment and VISTA plots has been widely employed, the approach may miss some architectural sequence features. For instance, in a VISTA plot comparing two genomes, one is marked as the reference sequence, and the plot is drawn relative to that sequence. Thus, insertions/deletions distinguishing the sequences are not easily seen. This is demonstrated in Figure 1 - in the VISTA plot, using *D. melanogaster *as the reference sequence, it is not possible to determine that the *D. pseudoobscura Sox21b *gene is expanded relative to the *D. melanogaster *homolog. However, our BLAST approach shows that this is the case. Also, while there are methods available to identify rearrangements during genome alignment [45], these are not readily presented using the VISTA plot format. Generally, examining VISTA plots of aligned sequence data may capture much of the important differences between orthologous regions of diverged species. However, some ultrastructural features of the sequences may be missed in some cases. Sliding-window BLAST-based procedures such as that presented here, or those implemented in the GATA software package [46], are likely to prove a worthwhile addition to the armory of those examining the causes and effects of DNA sequence differences between diverged species.

## Conclusion

We describe the use of a sliding-window BLAST-based approach to examine micro-scale genome architectural features. We almost certainly underestimate the actual number of such events occurring since the most recent common ancestor of these species, as in general there is considerable divergence between the genomes. Nevertheless, the microinversions we identify in this survey may be a particularly interesting class as they are conserved, and reside in introns or upstream of genes, and could have regulatory effects on gene expression and alternative exon splicing. We expect that microinversions will be fairly frequent in many organisms, not only *Drosophila*, and may be a particularly important source of genetic variation both among species and within populations.

## Materials and methods

### Genome sequences and annotation

The genome sequences of *D. melanogaster *(release 4.2.1) and *D. pseudoobscura *(release 1.04), and the annotation features for *D. melanogaster *(in GFF v.3 format) were downloaded from FlyBase [47]. Details of all the *D. melanogaster *genes were extracted from the GFF annotation files using a custom perlscript. Orthologous regions of *D. pseudoobscura *were identified via BLAST, using the standalone BLAST executable function blastall [48].

### Sliding-window BLAST comparison of orthologs

Release 4.2.1 of the *D. melanogaster *genome harbors 13,667 annotated protein-coding genes, each represented by a unique CG identifier. We identified 11,011 *D. melanogaster *protein-coding genes having orthologs in *D. pseudoobscura*. For each *D. melanogaster *gene sequence we scanned through the sequence in 15 bp steps, at each step BLASTing a 31 bp query sequence against the putative *D. pseudoobscura *ortholog. This was accomplished using a custom perlscript calling the standalone BLAST executable function bl2seq [48]. For each 31 bp *D. melanogaster *query sequence, we recorded the position, score, orientation and sequence of the best BLAST hit within the *D. pseudoobscura *ortholog. Only BLAST hits with scores above 45 were considered in further analyses. There were 5,738 orthologous pairs with at least two above threshold BLAST hits, and greater than 5% of the *D. melanogaster *gene sequence showing above threshold hits. Only these genes were retained for further analysis (Additional data file 1).

### Identification of structural features

A custom script written in the freely available statistical programming language R [49] was applied to each of the resulting sliding-window ortholog BLAST files. Inversions were recognized as at least two consecutive, above-threshold BLAST hits, where the *D. melanogaster *query sequences BLAST *D. pseudoobscura *in reverse orientation, and the order of the hits in the two genomes is reversed (that is, the *D. melanogaster *query sequences A-B-C-D-E are reverse complemented in *D. pseudoobscura*, and in reverse order E-D-C-B-A). We placed no restriction on the distance between BLAST hits defining a microinversion to avoid identifying only small events with high levels of nucleotide conservation throughout their length. This means that the threshold of nucleotide conservation required to detect a microinversion is not a constant across the genome. Large insertion/deletion events distinguishing the two genomes were also identified. To be detected, the endpoints of the BLAST hits flanking the insertion had to be separated by greater than 1 kb, and be 10 times more distant than the endpoints flanking the deletion. Plots for all 5,738 genes were manually checked to ensure the accuracy of our automatic scripts (Additional data file 6 [50]). Also, since we analyzed each gene independently, and genes can overlap in the *Drosophila *genome [27], we ensured that the inversion and insertion events we describe are unique.

### Testing for transposable element insertion

To test whether the large insertion/deletion events we observe are the result of TE insertion, we performed two tests. For those events where the insertion is in the *D. melanogaster *genome, we compared the position of each insertion with the positions of 6,013 TEs annotated in the *D. melanogaster *genome [23,24]. No corresponding database exists for *D. pseudoobscura*. Second, using BLAST we compared each insertion sequence to a set of TE sequences identified in *Drosophila*. These sequences are present in the file 'D_mel_transposon_sequence_set.fasta' (version 9.4.1) available from the BDGP natural transposable element project website [51].

### Confirmation of microinversion events

To ensure that inferred inversion events are not generally the result of genome assembly errors we designed 1 kb PCR amplicons about three of the inversion events: CG3578_inv1, CG3936_inv4, and CG32139_inv1. Products were amplified in the fly strains used for genome sequencing, that is, *D. melanogaster *stock number 2057 (Bloomington stock Center) or *D. pseudoobscura *stock number 14011-0121.94 (Tucson Drosophila species stock center). Accuracy of the genome assemblies was confirmed via dideoxy sequencing. PCR/sequencing oligos are available in Additional data file 7.

The orientation of a putatively inverted sequence in a genome assembly is likely correct if, relative to the flanking sequences, the orientation is preserved within one or more single shotgun sequencing reads. Hence, for the 54 intragenic microinversion events less than 500 bp in size in both species, we extracted the sequence of the inversion and the 100 bp flanking each breakpoint, and BLASTed against the appropriate genome shotgun trace archive database using Mega BLAST [52].

### GC content analysis

For each breakpoint of the 143 *D. melanogaster*-*D. pseudoobscura *microinversions we extracted a contiguous segment of 220 bp (200 bp flanking the breakpoint, and 20 bp internal to the inversion) from each species. For each species, independently for each breakpoint, across all sequences we calculate GC content for all overlapping 5 bp windows.

Conserved blocks were defined on the basis of the *D. melanogaster*-*D. pseudoobscura *sliding-window BLAST procedure described above. In the microinversions we identify, the average number of above-threshold BLAST hits per 100 bp of *D. melanogaster *sequence is 1.6. We therefore defined a conserved block as a sequence having at least 1.6 BLAST hits per 100 bp of *D. melanogaster *sequence. All of these hits must be between sequences having the same orientation in the two genomes. Furthermore, the 200 bp flanking each edge of the block must be free of above-threshold BLAST hits. Finally, the conserved blocks must be at least 200 bp in length in both species, and no part of the conserved blocks or flanking sequence can be exonic. Using these rules we identified 774 blocks of non-coding sequence conserved between *D. melanogaster *and *D. pseudoobscura *in the 93 genes harboring intronic microinversions. To examine GC content change across the boundaries of conserved and non-conserved sequence, using the 774 blocks we performed an analysis identical to that described for the microinversion breakpoints above.

### Phylogenetic distribution of microinversion events

For 15 intragenic microinversion events (CG2872_inv3, CG3578_inv1, CG3936_inv4, CG4220_inv1, CG4838_inv1, CG6464_inv1, CG9019_inv1, CG9623_inv1, CG11354_inv1, CG12154_inv1, CG12287_inv1, CG15455_inv1, CG31762_inv1, CG32139_inv1, CG33529_inv1), we identified the surrounding orthologous regions from 10 other *Drosophila *species using BLAST via the DroSpeGe website [53]. For each of the 15 regions we performed the sliding-window BLAST protocol described above, testing the *D. melanogaster *sequence and the *D. pseudoobscura *sequence independently against sequence from every other species. The presence of the standard (*D. melanogaster*-like) or inverted (*D. pseudoobscura*-like) sequence arrangement was recorded in each case.

Orthologous sequences were extracted from the following assemblies: dsim_wu050602 (*D. simulans*), dsec_br051028 (*D. sechellia*), dyak_caf051213 (*D. yakuba*), dere_caf051209 (*D. erecta*), dana_caf051209 (*D. ananassae*), dper_br051028 (*D. persimilis*), dmoj_caf051209 (*D. mojavensis*), dwil_caf060213 (*D. willistoni*), dvir_caf051209 (*D. virilis*), and dgri_caf051209 (*D. grimshawi*). The *D. simulans *and *D. yakuba *assemblies were provided by the Genome Sequencing Center, Washington University [54]. The *D. erecta*, *D. ananassae*, *D. mojavensis*, *D. virilis*, and *D. grimshawi *assemblies were provided by Agencourt Bioscience Corporation [55]. The *D. sechellia *and *D. persimilis *assemblies were provided by the Broad Institute [56]. The *D. willistoni *assembly was provided by the J. Craig Venter Institute [57].

## Additional data files

The following additional data files are available with the online version of this article. Additional data file 1 is a spreadsheet providing details of all 5,738 genes that are sufficiently conserved between *Drosophila melanogaster *and *D. pseudoobscura *to be tested. The number of microinversions and insertion/deletion events detected within each gene is also indicated. Additional data file 2 is a spreadsheet giving details of all 95 insertion/deletion events. Additional data files 3 and 4 are spreadsheets giving details of all 121 microinversions detected within genes, and all 22 microinversions detected upstream and downstream of genes, respectively. Additional data file 5 is a PDF showing histograms of the distance between the microinversion breakpoints and the nearest flanking exon for the 121 intragenic microinversions. Additional data file 6 is a zipped directory holding 5,738 PDFs, each showing a sliding-window *D. melanogaster*-*D. pseudoobscura *BLAST profile for a conserved pair of orthologs [50]. Additional data file 7 is a text file providing the sequences of the PCR/sequencing oligos used for microinversion validation.

## Supplementary Material

Additional data file 1 Tabulated data on all 5,738 tested orthologous gene pairs. Each row of the table represents a gene. The columns of the table are as follows: Column 1 ('CG') holds the CG identifier for the gene. Column 2 ('gene.name') holds the name of the gene, if any. Column 3 ('Dmel.geneINFO') gives the position of the gene within release 4.2.1 of the *Drosophila melanogaster *genome. Column 4 ('Dpse.geneINFO') gives the position of the gene within release 1.04 of the *D. pseudoobscura *genome. Column 5 ('num.exons') gives the number of exons in the gene. Column 6 ('Dmel.gene.bp') provides the length in base pairs of the gene in *D. melanogaster*. Column 7 ('BLASThit.bp') gives the amount of *D. melanogaster *sequence, in base pairs, included in the set of above-threshold sliding-window BLAST hits. Column 8 ('num.BLASThits') holds the number of above-threshold sliding-window BLAST hits. Column 9 ('num.inserts') gives the number of large insertion/deletions events detected in the gene. Column 10 ('num.inversions') gives the number of microinversion events detected in the gene. Click here for file

Additional data file 2 Tabulated data on all 95 large insertion/deletion (indel) events detected. Each row of the table represents an indel. The columns of the table are as follows. Column 1 ('CG') holds the CG identifier for the gene. Column 2 ('Dmel.chr') gives the *D. melanogaster *chromosome on which the gene resides. Columns 3, 4, and 5 ('Dmel.geneSTART', 'Dmel.geneSTOP', and 'Dmel.geneORIENT') give the gene start position, stop position, and orientation, respectively, in release 4.2.1 of the *D. melanogaster *genome assembly. Column 6 ('Dpse.chr') gives the *D. pseudoobscura *chromosome on which the gene resides. Columns 7, 8, and 9 ('Dpse.geneSTART', 'Dpse.geneSTOP', and 'Dpse.geneORIENT') give the gene start position, stop position, and orientation, respectively, in release 1.04 of the *D. pseudoobscura *genome assembly. Columns 10 and 11 ('Dmel.insSTARTREL' and 'Dmel.insSTOPREL') give the position of the indel relative to the start of the host *D. melanogaster *gene. Columns 12 and 13 ('Dmel.insSTART.genome' and 'Dmel.insSTOP.genome') give the position of the indel in release 4.2.1 of the *D. melanogaster *genome assembly. Columns 14 and 15 ('Dpse.insSTARTREL' and 'Dpse.insSTOPREL') give the position of the indel relative to the start of the host *D. pseudoobscura *gene. Columns 16 and 17 ('Dmel.LEN' and 'Dpse.LEN') provide the lengths of the insertion/deletion in each genome. Column 18 ('insert.species') notes the species harboring the insert. Column 19 ('insert.amount') notes the amount of DNA inserted in base pairs. For those indels showing the insertion in *D. melanogaster *column 20 ('Dmel.insert.annot.TE') notes if the insertion overlaps the position of a known transposable element in the *D. melanogaster *genome. Column 21 ('insert.BLAST') shows the type of transposable element, if any, the inserted sequence BLASTs to. Click here for file

Additional data file 3 Tabulated data on all 121 microinversions detected by scanning regions within genes. Each row of the table represents a microinversion. The columns of the table are as follows. Column 1 ('CG') holds the CG identifier for the gene. Column 2 ('Dmel.chr') gives the *Drosophila melanogaster *chromosome on which the gene resides. Columns 3, 4, and 5 ('Dmel.geneSTART', 'Dmel.geneSTOP', and 'Dmel.geneORIENT') give the gene start position, stop position, and orientation, respectively, in release 4.2.1 of the *D. melanogaster *genome assembly. Column 6 ('Dpse.chr') gives the *D. pseudoobscura *chromosome on which the gene resides. Columns 7, 8, and 9 ('Dpse.geneSTART', 'Dpse.geneSTOP', and 'Dpse.geneORIENT') give the gene start position, stop position, and orientation, respectively, in release 1.04 of the *D. pseudoobscura *genome assembly. Column 10 ('inv.num') applies an arbitrary number to each microinversion so independent events within a single gene can be distinguished. Columns 11 and 12 ('Dmel.invSTARTREL' and 'Dmel.invSTOPREL') give the positions of the microinversion breakpoints relative to the start of the host *D. melanogaster *gene. Columns 13 and 14 ('Dmel.invSTART.genome' and 'Dmel.invSTOP.genome') give the positions of the microinversion breakpoints in release 4.2.1 of the *D. melanogaster *genome assembly. Column 15 ('Dmel.exon.overlap') is a 0/1 vector indicating whether the microinversion overlaps with an annotated *D. melanogaster *exon. Column 16 ('Dmel.gene.overlap') gives the identifier of the nested gene an inversion overlaps. Column 17 ('Dmel.invLEN') is the length of the microinversion in *D. melanogaster*. Columns 18 and 19 ('Dpse.invSTARTREL' and 'Dpse.invSTOPREL') give the positions of the microinversion breakpoints relative to the start of the host *D. pseudoobscura *gene. Column 20 ('Dpse.invLEN') is the size of the microinversion in *D. pseudoobscura*. Column 21 ('num.Dmel.introns') is the number of introns in the *D. melanogaster *host gene. Column 22 ('Dmel.invINTRON') is the number of the intron within which the microinversion resides, column 23 ('Dmel.invINTRON.size') is the size of that intron, and column 24 ('Dmel.invINTRON.sizerank') is the ranked size of that intron, with a 1 indicating the microinversion is present within the largest intron. Columns 25 and 26 ('Dmel.invINTRON.leftdist' and 'Dmel.invINTRON.rightdist') are distances between the 5'-breakpoint and the 3'-breakpoint, respectively, of the microinversion and the nearest flanking exon. Click here for file

Additional data file 4 Tabulated data on all 22 microinversions detected by scanning 2 kb regions upstream and downstream of each conserved orthologous gene pair. Each row of the table represents a microinversion. The columns of the table are as follows. Column 1 ('CG') holds the CG identifier for the gene. Column 2 ('Dmel.chr') gives the *D. melanogaster *chromosome on which the gene resides. Columns 3, 4, and 5 ('Dmel.geneSTART', 'Dmel.geneSTOP', and 'Dmel.geneORIENT') give the gene start position, stop position, and orientation, respectively, in release 4.2.1 of the *D. melanogaster *genome assembly. Column 6 ('Dpse.chr') gives the *D. pseudoobscura *chromosome on which the gene resides. Columns 7, 8, and 9 ('Dpse.geneSTART', 'Dpse.geneSTOP', and 'Dpse.geneORIENT') give the gene start position, stop position, and orientation, respectively, in release 1.04 of the *D. pseudoobscura *genome assembly. Column 10 ('inv.num') gives an arbitrary number to each microinversion so independent events within a single gene can be distinguished. Columns 11 and 12 ('Dmel.invSTARTREL' and 'Dmel.invSTOPREL') give the positions of the microinversion breakpoints relative to the start of the host *D. melanogaster *gene. Columns 13 and 14 ('Dmel.invSTART.genome' and 'Dmel.invSTOP.genome') give the positions of the microinversion breakpoints in release 4.2.1 of the *D. melanogaster *genome assembly. Column 15 ('Dmel.invPOS.relgene') indicates whether the microinversion is 5' or 3' of the test gene. Column 16 ('Dmel.exon.overlap') is a 0/1 vector indicating whether the microinversion overlaps with an annotated exon in *D. melanogaster*. Column 17 ('Dmel.gene.overlap') gives the identifier of the nested gene an inversion overlaps. Column 18 ('Dmel.invLEN') is the length of the microinversion in *D. melanogaster*. Columns 19 and 20 ('Dpse.invSTARTREL' and 'Dpse.invSTOPREL') give the positions of the microinversion breakpoints relative to the start of the host *D. pseudoobscura *gene. Column 21 ('Dpse.invLEN') is the size of the microinversion in *D. pseudoobscura*. Click here for file

Additional data file 5 For all 121 microinversions identified by scanning within genes, the distance between each breakpoint and the closest flanking exon was extracted, and divided by the size of the host intron. The plots are histograms of the 121 weighted distances, considering the 5'- and 3'-breakpoints separately. To test the distances against a uniform distribution we used a two-sided Kolmogorov-Smirnov test, and the results are presented above the plots. Click here for file

Additional data file 6 This file is a zipped archive of the sliding-window BLAST profiles (as PDFs) for all 5,738 orthologous gene pairs tested. The file is available at [50]. The name of the plot is the CG identifier for the gene. We stepped through *D. melanogaster *gene in 15 bp increments, and at each position BLASTed a 31 bp segment against the *D. pseudoobscura *ortholog. Each line represents a BLAST hit with a score above 45, the endpoints show the position of the hit in each genome, and the color of line represents the orientation of the hit (black = same sequence orientation in each genome, red = different orientations in each genome). Click here for file

## Figures and Tables

**Table 1:** Distribution of fine-scale structural features across chromosome arms

	Number of orthologous pairs		
	
Chromosome arm*	Tested^†^	Harboring microinversions^‡^	Harboring intragenic indels^‡^
*X*	880	24 (0.111)	12 (0.889)
*2L*	997	34 (0.003)	19 (0.294)
*2R*	1,120	7 (<0.001)	15 (0.805)
*3L*	1,177	21 (0.752)	22 (0.279)
*3R*	1,560	26 (0.464)	18 (0.297)
All 5 major arms	5,734	112	86

*The chromosome arms are given the *D. melanogaster *designations, *X*, *2L*, *2R*, *3L*, and *3R*. These arms are known to be orthologous to *D. pseudoobscura *arms, *XL*, *4*, *3*, *XR*, and *2*, respectively [58]. ^†^The number of conserved orthologs residing on each arm. ^‡^Values in parentheses are *P *values from a two-sided Binomial test of whether the number of event-harboring orthologs per arm differs from expectation. For each test, the number of trials equals the number of conserved orthologs per arm, the number of successes equals the number of event-harboring genes per arm, and the probability of success is equal to the total number of event-harboring genes detected divided by the total number of conserved orthologs tested (5,738).
