# Supplementary material for: Fine scale structural variants distinguish the genomes of Drosophila melanogaster and D. pseudoobscura
Source: Genome Biol. 2006 Jul 27;7(7):R67. doi: 10.1186/gb-2006-7-7-r67 (PMC1779558; doi:10.1186/gb-2006-7-7-r67)
Supplement: Additional data file 5 — For all 121 microinversions identified by scanning within genes, the distance between each breakpoint and the closest flanking exon was extracted, and divided by the size of the host intron. The plots are histograms of the 121 weighted distances, considering the 5'- and 3'-breakpoints separately. To test the distances against a uniform distribution we used a two-sided Kolmogorov-Smirnov test, and the results are presented above the plots. [file gb-2006-7-7-r67-S5.pdf]

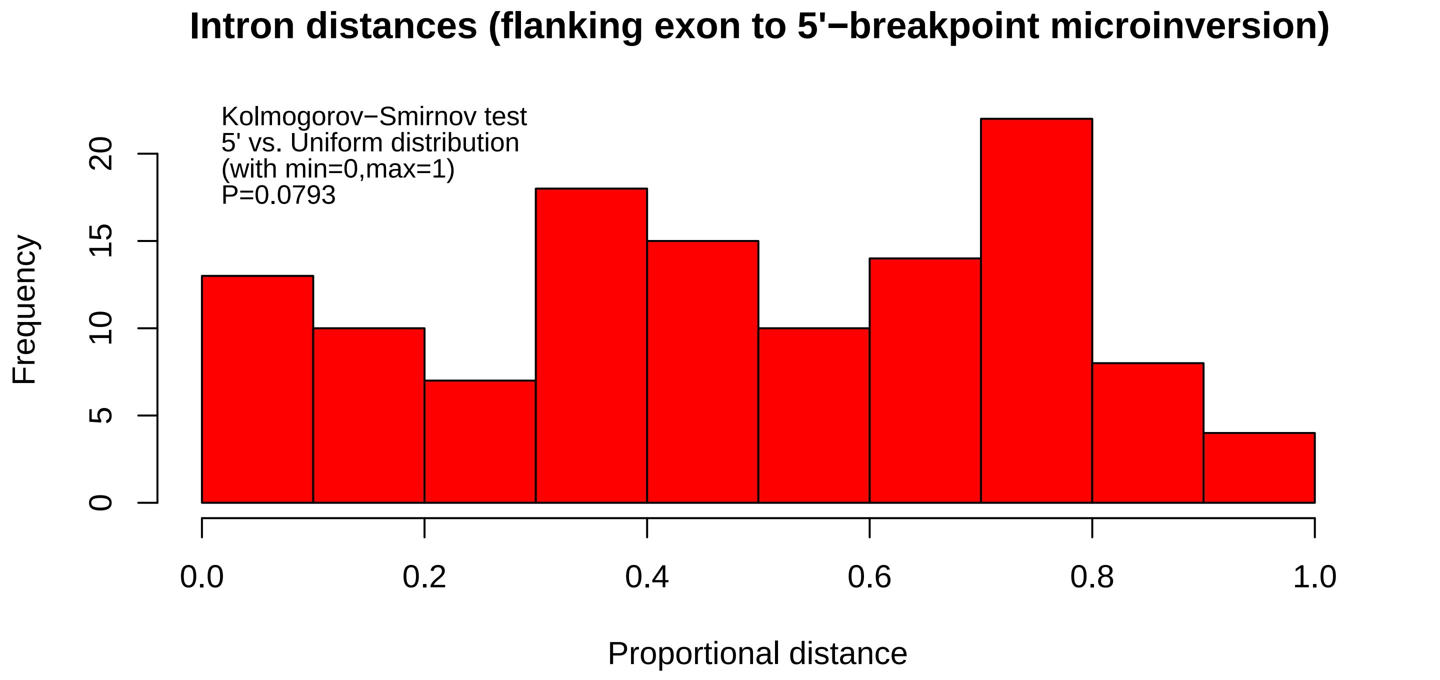
Intron distances (flanking exon to 5'−breakpoint microinversion)
Kolmogorov−Smirnov test
5' vs. Uniform distribution
(with min=0,max=1)
P=0.0793
Frequency
0
5
10
15
20
0.0
0.2
0.4
0.6
0.8
1.0
Proportional distance

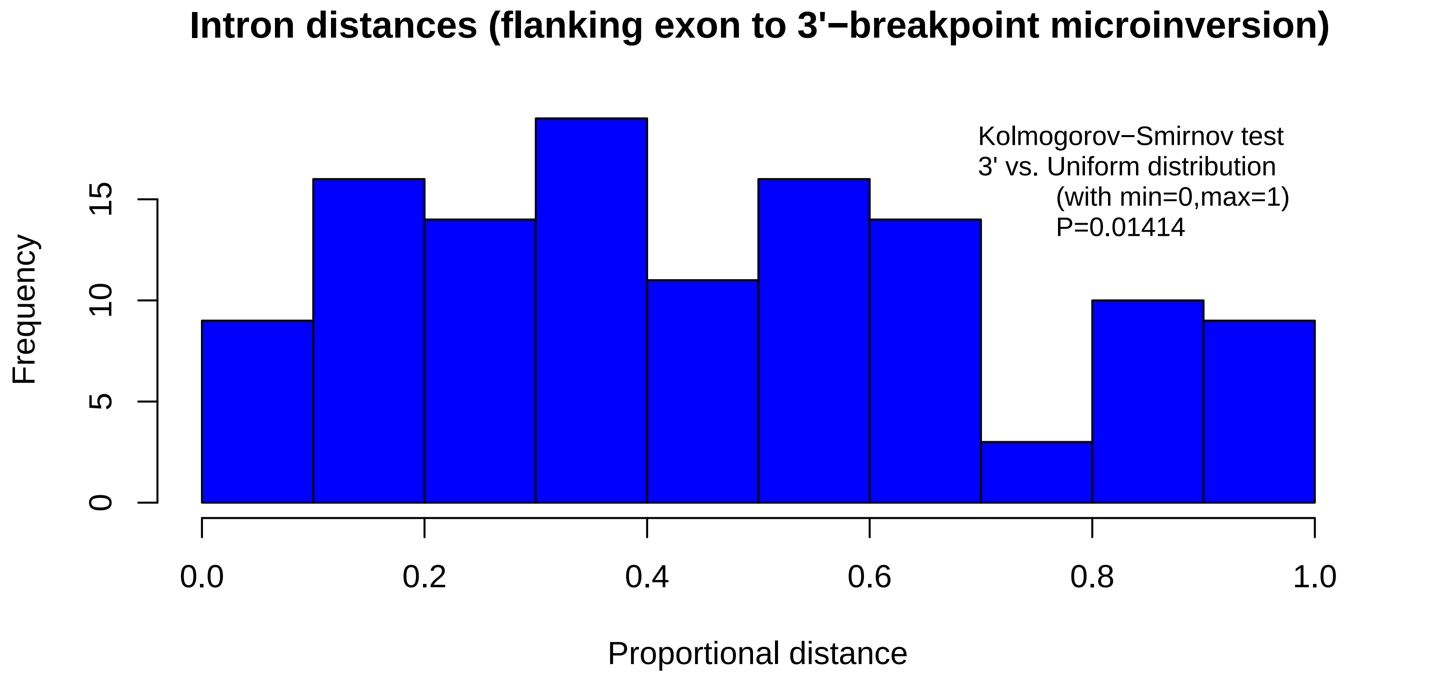
Intron distances (flanking exon to 3'−breakpoint microinversion)
Kolmogorov−Smirnov test
3' vs. Uniform distribution
(with min=0,max=1)
P=0.01414
Frequency
0
5
10
15
0.0
0.2
0.4
0.6
0.8
1.0
Proportional distance
